# Subtype Diagnosis of Primary Aldosteronism: Is Adrenal Vein Sampling Always Necessary?

**DOI:** 10.3390/ijms18040848

**Published:** 2017-04-17

**Authors:** Fabrizio Buffolo, Silvia Monticone, Tracy A. Williams, Denis Rossato, Jacopo Burrello, Martina Tetti, Franco Veglio, Paolo Mulatero

**Affiliations:** 1 Division of Internal Medicine and Hypertension Unit, Department of Medical Sciences, University of Torino, Via Genova 3, 10126 Torino, Italy; fabrizio.buffolo@gmail.com (F.B.); silvia.monticone@unito.it (S.M.); tracyannwilliams48@gmail.com (T.A.W.); jacopo.burrello@gmail.com (J.B.); tetti.martina@gmail.com (M.T.); franco.veglio@unito.it (F.V.); 2 Medizinische Klinik und Poliklinik IV, Klinikum der Ludwig-Maximilians-Universität München, 81377 Munich, Germany; 3 Service of Radiology, University of Torino, 10126 Torino, Italy; denisrossato@gmail.com

**Keywords:** aldosterone, primary aldosteronism, adrenal vein sampling, cosyntropin stimulation, aldosterone producing adenoma, bilateral adrenal hyperplasia

## Abstract

Aldosterone producing adenoma and bilateral adrenal hyperplasia are the two most common subtypes of primary aldosteronism (PA) that require targeted and distinct therapeutic approaches: unilateral adrenalectomy or lifelong medical therapy with mineralocorticoid receptor antagonists. According to the 2016 Endocrine Society Guideline, adrenal venous sampling (AVS) is the gold standard test to distinguish between unilateral and bilateral aldosterone overproduction and therefore, to safely refer patients with PA to surgery. Despite significant advances in the optimization of the AVS procedure and the interpretation of hormonal data, a standardized protocol across centers is still lacking. Alternative methods are sought to either localize an aldosterone producing adenoma or to predict the presence of unilateral disease and thereby substantially reduce the number of patients with PA who proceed to AVS. In this review, we summarize the recent advances in subtyping PA for the diagnosis of unilateral and bilateral disease. We focus on the developments in the AVS procedure, the interpretation criteria, and comparisons of the performance of AVS with the alternative methods that are currently available.

## 1. Introduction

Primary aldosteronism (PA) is the most frequent cause of endocrine hypertension, with a prevalence of around 6% in patients from primary care and up to 30% in referral units [1,2]. The majority of affected patients have a sporadic form, either bilateral adrenal hyperplasia (BAH) or an aldosterone producing adenoma (APA), that accounts for over 90% of all cases. Other forms of sporadic PA are unilateral adrenal hyperplasia and adrenal producing carcinoma; familial forms account for less than 6% of all cases of PA [3].

Compelling evidence indicates that an excess of aldosterone exerts a detrimental role on the cardiovascular system and affected patients display an increase in cardio and cerebrovascular complications compared to patients with essential hypertension with similar risk profiles [2,4,5]. For this reason, an early diagnosis and specific treatment are important to revert target organ damage and prevent cardiovascular events. The diagnosis of PA is made by a multistep process: (I) screening test, (II) confirmatory testing, and (III) subtype differentiation [6]. The last step is of fundamental importance to distinguish between unilateral and bilateral PA, which require different therapeutic approaches (unilateral laparoscopic adrenalectomy or medical therapy with a mineralocorticoid receptor antagonist). According to the Endocrine Society (ES) Guideline, a subtype diagnosis comprises adrenal CT scanning and adrenal venous sampling (AVS), a demanding procedure that requires an expert and dedicated radiologist. Despite recent expert consensus, intended to address several issues concerning performance and interpretation [7,8], AVS remains a poorly standardized procedure across centers. This issue, together with reports [9,10] indicating high complication rates, has fostered several attempts to develop clinical and biochemical prediction models of unilateral PA, “ancillary” hormonal testings, or innovative targeted imaging techniques to overcome the need for AVS, at least in a proportion of patients, and to use this procedure in patients with a high probability of unilateral PA. In this manuscript, we critically review AVS performance in comparison with alternative tests or clinical criteria in PA subtype diagnosis.

## 2. Comparison of AVS with Imaging Techniques

Adrenal CT scanning and magnetic resonance (MR) are the imaging techniques more commonly used to evaluate adrenal morphology in PA patients [6]. According to the ES Guideline, adrenal CT scanning is mandatory before AVS to evaluate adrenal morphology, abdominal venous drainage, and exclude an adrenal carcinoma, but its sensitivity and specificity for subtype differentiation is poor and does not exceed 87% and 71%, respectively, even when evaluated by an expert and dedicated radiologist [11]. Although early studies suggested that imaging techniques were useful to locate APAs [12], it became evident that CT and MR results were often discordant with AVS for the subtype diagnosis of PA. The diagnostic accuracy of imaging techniques is hampered by intrinsic limitations of the procedures, such as a suboptimal spatial resolution and a lack of functional data, that result in a failure to identify micro APAs (less than 1 cm) or unilateral adrenal hyperplasia, and to differentiate between APA and non-functioning adrenal incidentaloma [13,14].

An extensive systematic review [15] evaluating 950 patients from 38 different studies, reported that CT/MR-based diagnoses were discordant with AVS results in 37.8% of patients. Following a diagnosis by CT/RM imaging, 14.6% of patients would have been incorrectly identified as unilateral PA and adrenalectomized (compared to the AVS diagnosis of bilateral aldosterone production); CT/MR imaging would have also incorrectly diagnosed bilateral PA in 19.1% of patients (with AVS demonstrating unilateral aldosterone production), thereby negating the possibility of a cure for PA and hypertension. Even more importantly, an adrenalectomy would have been performed on the wrong side in 3.9% of cases (i.e., contralateral to the site of aldosterone hypersecretion). When AVS demonstrated unilateral aldosterone secretion in disagreement with the CT/RM results, imaging techniques showed bilateral abnormalities in 39.9% of patients, no abnormalities in 30.9% of patients and abnormalities in the opposite side compared to the AVS results in 17.0% of cases (in the remaining cases, the exact result of the imaging technique was indicated as bilateral forms without further indications) [15].

AVS is therefore considered as the gold standard test for PA subtype diagnosis; however, the evidence for its superiority over adrenal imaging in terms of the clinical outcomes after an adrenalectomy is limited. To this aim, Dekkers et al. designed a three-year randomized control trial aimed at comparing two groups of CT- and AVS-based management in patients with confirmed PA [16]. A total of 184 PA patients completed the follow up (92 received CT-based treatment and 92 AVS-based treatment) and were included in the study. The patients were evaluated according to an intention-to-diagnose analysis and, after one year, there was no difference between the two groups in the intensity of drug treatment required to obtain a target blood pressure, biochemical cure rate, health-related quality of life, and adverse events. Of note, after a unilateral adrenalectomy, a total of 14 patients (15%), nine in the CT subgroup (20%) and five (11%) in the AVS subgroup (*p* = 0.25), showed persistent PA; the authors concluded that both CT scanning and AVS are imperfect tests to identify the patients who are suitable for an adrenalectomy. Some concerns about the design of the study and the application of the results to all patients with PA were raised [17,18]. The inclusion criteria comprised a confirmed PA diagnosis and resistant hypertension or spontaneous/diuretic-induced hypokalemia. Using these criteria, patients with the most severe PA and thus with a high probability of unilateral PA were selected [18]. In addition, the study was designed to detect a 0.8 difference in the defined daily dose between the two groups, but the real power of the study was inferior if only the patients undergoing an adrenalectomy were considered [18]. It has been shown that a CT-based and AVS-based diagnosis corresponds in 62% of cases [15]: therefore, the effective number of candidate patients for an adrenalectomy in which a different diagnostic approach would have led to a different outcome, is not likely to be greater than 35 individuals. Finally, a previous study [19] reported that an adrenalectomy could also improve blood pressure levels in a significant proportion of BAH patients, reducing the possibility to detect outcome differences in a relatively small cohort of patients. In conclusion, the low power of the study does not allow one to draw any definitive conclusion or to generalize the results to all PA patients. Further studies are warranted, and in the meanwhile, affected patients should not be denied an AVS procedure, which remains the most reliable means to refer them for a unilateral adrenalectomy or medical therapy.

## 3. Patients in Which AVS Can Be Avoided

Despite being the “gold standard” test to distinguish between unilateral and bilateral PA [6], AVS remains a costly and challenging procedure, and is only routinely available in some tertiary referral centers worldwide [20].

ES guidelines [6], despite the low amount of available evidence, suggest that in patients with the following characteristics: (I) younger than 35 years, (II) severe PA phenotype (e.g., plasma aldosterone concentration (PAC) > 30 ng/dL and spontaneous hypokalemia), and (III) CT scan showing a unilateral adenoma with a normal contralateral adrenal gland, AVS can be avoided and it is possible to directly proceed to a unilateral adrenalectomy [6,21,22].

However, the number of patients with PA who could avoid AVS according to these clinical criteria is only about 10% [23]. For these reasons, considerable effort has been devoted to identify sensitive and specific scores or ancillary hormonal tests to predict the lateralization of aldosterone production and safely refer patients with PA to surgery without performing AVS or to limit the number of AVS procedures. Küpers et al. reported that the combination of a solitary nodule on a CT scan and low serum potassium (<3.5 mmol/L) or an estimated glomerular filtration rate ≥100 mL/min/1.73 m^2^, has a 100% specificity for APA diagnosis [23]. However, the number of patients included in the analysis was relatively small and subsequent studies on larger cohorts [21,24] did not confirm the results. The sensitivity and specificity of the currently available clinical scores used to predict unilateral PA are summarized in (Table 1).

When a CT scan, evaluated by an experienced radiologist, displays bilaterally normal adrenal glands, AVS demonstrates bilateral aldosterone secretion in 95% of patients [11]. The treatment of these patients with mineralocorticoid receptor antagonist (MRA) without AVS performance, is a reasonable option, especially in centers where AVS is not readily available.

A recent study [30] evaluated the usefulness of the administration of intravenous ACTH (1-24) before measuring the plasma aldosterone concentration in the peripheral vein. An ACTH stimulation test (with a cut off value of 37.9 ng/dL for plasma aldosterone level) predicted the presence of unilateral PA with a sensitivity and a specificity of 91.3% and 80.6%, respectively.

18-hydroxycortisol and 18-oxocortisol, also known as hybrid steroids, are synthesized by aldosterone synthase using 11-deoxycortisol as a substrate (although 18OHF can also be produced by 11β-hydroxylase), and have been detected at very high levels in some familial forms [37,38]. 18-hydroxycorticosterone is a minor steroid synthesized by aldosterone synthase from deoxycorticosterone; 18-hydroxycorticosterone is higher in individuals with APA than in BAH patients and it has been proposed, since the late 1970s, as a biochemical marker to distinguish between unilateral and bilateral aldosterone production. In support of this hypothesis, a further study demonstrated that serum 18-hydroxycorticosterone, serum and urinary 18-hydroxycortisol, and 18-oxocortisol are higher in patients with APA than in patients with BAH [33]. Moreover, plasma 18-oxocortisol (measured by LC/MS/MS) above 6.1 ng/dL, together with a plasma aldosterone concentration higher than 32.7 ng/dL, was found in 84% of APA patients, but in none of the BAH cases [34]. In a retrospective study (79 patients with unilateral PA), a peripheral venous steroid biomarker panel correctly classified the APA genotype in 92% of cases [39]. Patients with APA carrying *KCNJ5* mutations displayed markedly elevated concentrations of the hybrid steroids 18-oxocortisol and 18-hydroxycortisol in the peripheral plasma, which is presumably caused by the underlying histology of these adenomas with a predominant composition of zona fasciculata-like cells and an increased expression of *CYP17A1* [40,41]. APA carrying *KCNJ5* mutations [42] account for nearly 40% of sporadic adenomas [43], a frequency that increases to 73% in populations from East Asia [44]. This high prevalence contributes to the potential utility of steroid profiling for subtype differentiation in PA and could be responsible for the high success rate of 18-oxocortisol measurements for distinguishing patients with APA in a study from Japan, where the prevalence of KCNJ5 mutations is notably high [34].

Similarly, Eisenhofer et al. [32] proposed that the use of the steroid profiling in peripheral blood could be useful to reduce the number of AVS, through the identification of patients with a high probability of BAH, which were correctly identified in 76% of the cases.

Many alternative imaging tests have been proposed for the subtype diagnosis of PA, even though none of them currently exhibit an adequate sensitivity and specificity to be relied on to refer PA patients for a unilateral adrenalectomy. 6β-131iodomethyl-19-norcholesterol (NP-59) scanning, performed under dexamethasone suppression, may correlate the anatomical findings with functional features. However, it is not sensitive enough to detect a small APA (<1.5 cm), it is not widely available and, therefore, does not play a significant role in subtype differentiation [6].

^11^C-metomidate positron emission tomography (PET)-CT scanning has been recently proposed as an alternative test for PA subtyping, with a sensitivity of 76% and a specificity of 87% compared to AVS [36]. Metomidate is a potent inhibitor of CYP11B1 (11β-hydroxylase), which is involved in the last step of cortisol synthesis, and CYP11B2 (aldosterone synthase), the main regulator of aldosterone synthesis. The administration of exogenous glcorticoid (dexamethasone) before the procedure significantly reduces CYP11B1 expression in a normal adrenal gland, increasing the “contrast” between the APA and normal adrenal tissue [45]. The major limitation of a wide use of this procedure is the necessity of an on-site cyclotron, because of the very short half-life of carbon-11 [36]. To this regard, some authors are currently exploring alternative tracers with a longer half-life [46]. Abe et al. [47] recently synthesized a novel radiolabeled tracer (^18^F-CDP2230), displaying a lower affinity for CYP11B2 and CYP11B1 compared to ^11^C-metomidate, but a relatively higher selectivity for CYP11B2 over CYP11B1. The higher selectivity for CYP11B2, the longer half-life, and the absence of the requirement for dexamethasone pretreatment, are the three main advantages of ^18^F-CDP2230 over metomidate. Human in vivo studies are needed to further evaluate its specificity and sensitivity in PA subtyping.

The presence of familial hyperaldosteronism type I and III, in which the disease is always bilateral, should be ruled out before AVS. The diagnosis of familial hyperaldosteronism type I made by the demonstration of the hybrid *CYP11B1/CYP11B2* gene, should be considered in all PA patients with an early onset of PA (<20 years), a family history of PA, or a family history of stroke at a young age [6]. Familial hyperaldosteronism type III, a rare but particularly severe form of PA, due to germline *KCNJ5* mutations [42], should be considered in all PA patients with an onset at a very young age (<20 years) [6]. Recently, germline mutations in the *CACNA1H* gene, encoding for the alpha subunit of a L-type voltage-gated calcium channel (Cav3.2), were discovered to be responsible for a novel form of familial hyperaldosteronism (type IV) with an incomplete penetrance. However, Zennaro et al. [48] reported a single case of a *CACNA1H* germline mutation identified in one patient who underwent an adrenalectomy, followed by a complete biochemical and clinical response (AVS was not performed). To this regard, further insights into the familial hyperaldosteronism type IV spectrum are currently needed to define the best diagnostic and therapeutic approach in this familial form of PA.

## 4. AVS Methodology

AVS is performed via a percutaneous femoral vein approach and the adrenal veins are cannulated using a fluoroscopy guide (Supplementary multimedia file). The left adrenal vein is usually easily cannulated, because it generally drains directly into the left renal vein [49], while the cannulation of the shorter and smaller right adrenal vein, which usually drains directly into the inferior vena cava, can be difficult. Despite being a challenging procedure, the complication rate associated with AVS is very low (0.61%) [20]; adrenal haemorrhage, which is the most serious complication, has a favourable outcome in the majority of patients, causing minor or no permanent effects on adrenal function [50]. Other AVS protocols are described below.

## 5. Bilaterally Simultaneous vs. Sequential Cannulation

The main proposed advantage of the simultaneous cannulation of the adrenal veins is to avoid oscillations of aldosterone and cortisol secretion, which could artificially modify the gradient of aldosterone production between the two adrenals if the procedure is performed under basal conditions [51]. On the other hand, simultaneous AVS is more invasive (requiring the insertions of two catheters) and demanding than the sequential technique. Recently, a retrospective study [52] comparing simultaneous and sequential AVS (with a 5 min time-gap between cannulations) under basal conditions, demonstrated no significant differences in the selectivity and lateralization indices. However, whether long time-gaps (e.g., during a difficult cannulation of one adrenal gland) could significantly modify the AVS results is still unknown. This study demonstrated that sequential cannulation displays the same accuracy compared with simultaneous cannulation, even in an unstimulated procedure, as long as the time between the cannulation of the two adrenal veins remains relatively short.

## 6. Segmental AVS

Some authors propose segmental AVS as a way to refer PA patients for a selective nodulectomy, instead of the total resection of the adrenal gland [53]. This is particularly relevant to enable the surgical cure of patients with bilateral APA and in patients with APA in the remaining adrenal gland after a contralateral adrenalectomy [53].

In segmental AVS, samples are collected from tributaries of the central adrenal vein and compared to each other to provide more detailed information regarding the location of the secreting nodule within the gland. The main disadvantages of this technique are that segmental AVS is technically even more demanding, time-consuming and expensive [53]. Furthermore, in two case series, an extravasation of the iodine contrast occurred in 12.6% [53] and in 16% [54] of patients undergoing segmental AVS, making this complication more common than in central AVS [53].

## 7. Cosyntropin Stimulation

ACTH 1-24 is a synthetic derivate of the adrenocorticotropic hormone (ACTH), whose infusion during the AVS procedure was introduced by Melby et al. in 1967 [55]. Two major protocols of ACTH (1-24) administration are currently used: (I) ACTH (1-24) continuous infusion (50 µg/h, starting 30 min before the first sampling), and (II) ACTH bolus (250 µg [10 IU]).

The main advantage of the ACTH (1-24) infusion is to maximise the cortisol gradient between the adrenal and peripheral veins, to minimise aldosterone fluctuations, and to maximise aldosterone production from an APA [8,13]. Furthermore, ACTH (1-24) infusion is necessary for patients at a high risk of allergic reaction, in which steroidal prophylaxis is required, in patients with subclinical hypercortisolism (to stimulate cortisol production from the contralateral gland, which is expected to be suppressed), and when the AVS procedure is not performed in the early morning, when cortisol secretion in higher. Moreover, in centres with low rates of success of adrenal cannulation, the ACTH (1-24) infusion can significantly improve the percentage of successful procedures [56,57,58].

The main criticism of the ACTH (1-24) infusion is that it could potentially stimulate aldosterone secretion from the contralateral adrenal gland (in respect to an APA) and therefore reduce the lateralisation index [59]. A multicenter retrospective study [58] showed a concordant diagnosis between ACTH (1-24) stimulated and basal AVS in 88% of cases with continuous infusion protocol and 78% with ACTH (1-24) intravenous bolus. Interestingly, the reproducibility of the diagnosis was lower with more permissive criteria of lateralisation, increasing the percentage of patients potentially susceptible to inappropriate treatment [58]. El Ghorayeb et al. found that basal and post-ACTH AVS results were discordant in 28% of cases, with the great majority of lateralized procedures under basal conditions becoming bilateral after the administration of ACTH (1-24). However, the results might have been affected by the fact that the authors chose a higher LI cut-off for the ACTH-stimulated procedures compared to the basal ones (LI ≥ 4 and LI ≥ 2, respectively), despite a slight decrease in the median LI after ACTH (from 7 to 6.7) [56]. In addition, Wolley et al. suggested that ACTH (1-24) administration might be useful in those cases with bilaterally suppressed aldosterone production (i.e., an IL ratio lower than the peripheral on both sides) [57].

In conclusion, cosyntropin-stimulated AVS displays a similar performance and diagnostic accuracy compared with unstimulated AVS. Cosyntropin infusion could be the preferred strategy in centres with a lower success rate of cannulation.

## 8. Improving the Success Rate

A wealth of studies [11,13,20,49,60,61] have demonstrated that an expert and dedicated radiologist is a key factor for significantly improving sampling success. Therefore, gathering AVS procedures in one single centre per geographical area is important to further reduce the number of unsuccessful studies.

Other than ACTH (1-24) administration, another strategy to improve performances in centres with a low success rate is through the use of a rapid cortisol assay, performed during the AVS procedure. Measurements of cortisol can be done in the central laboratory of the hospital [62,63,64] or inside the radiology room using a benchtop analyser [65]. The latter method, performed with a reliable cortisol immunofluorimetric assay [65] or with immunochromatography and gold nanoparticles [66], more rapidly provide information to the radiologist on the position of the catheter tip. Rapid cortisol measurements enable the radiologist further attempts to correctly cannulate the adrenal veins, and allow self-training of the radiologist to progressively increase the percentage of successful procedures [62].

Dyna computed tomography performed during the AVS procedure, is an alternative method to evaluate the correct catheter tip positioning and was recently proposed by Chang et al. [67], allowing a correct repositioning of the catheter tip in 12% of cases.

## 9. AVS Interpretation Criteria

The selectivity index (SI) is defined as the cortisol ratio between an adrenal vein and a peripheral vein and it is used to confirm the adequacy of adrenal vein catheterization (Table 2). Most centres use an SI cut-off of at least two to three under basal conditions and three to five under ACTH (1-24) stimulation, with a minority of the centres using more permissive SI criteria under a basal condition (≥1.1 or 1.36) [20].

In order to identify the best SI cut-off value in the basal condition, Mulatero et al. [68] evaluated the concordance of the AVS results in patients who underwent two AVS procedures on two separate occasions, because the first one was unsatisfactory. The most permissive SI criteria (SI ≥ 1.1) provided a concordant diagnosis in only 35% of patients and intermediate criteria (SI ≥ 2.0) in only 50% of patients. An SI > 2.7 was necessary to achieve 100% reproducibility in the same patients.

Considering an SI cut-off of five (under ACTH (1-24) stimulation) as the reference standard, Mailhot et al. [69] investigated the best SI cut-off value for AVS performed under a basal condition. An SI cut-off of two provided the highest sensitivity (70.8%) with 100% specificity. Interestingly, they proposed the use of the aldosterone ratio (defined as the ratio between the aldosterone levels in the adrenal vein over the peripheral vein) combined with the cortisol ratio to further improve the SI sensitivity in the basal condition. AVS was considered satisfactory if at least one of the two ratios met the SI criteria. An SI cut-off of two for both (aldosterone and cortisol) ratios provided the greatest sensitivity (88.3% of procedures) with a 100% specificity.

The lateralization index (LI) is defined as the ratio between the aldosterone/cortisol level in the dominant side and the aldosterone/cortisol level in the contralateral gland, and is used to determine the final subtype diagnosis in the majority of centres (Table 2). Currently, there is not a wide consensus on the optimal LI cut-off to better differentiate between unilateral and bilateral forms. Although a LI ≥ 4 is deemed to be diagnostic of unilateral PA in most units, some centres adopt more permissive criteria (LI > 3 more commonly and LI > 2 in few centres).

In a recent informative study, AVS performed in patients with a positive screening test for PA but a negative confirmatory testing, demonstrated that eight out of 40 patients had an LI comprised between two and four, and no patients showed an LI greater than 4 suggesting that 2 ≤ LI ≤ 4 should be cautiously interpreted [70].

In some centres, an ipsilateral ratio (IR) > 2, together with a contralateral aldosterone suppression (defined as CL ratio (CR) < 1, Table 2), are used instead of LI to determine whether the disease is unilateral [71]. However, the clinical significance of contralateral suppression and its impact on postoperative outcomes after a unilateral adrenalectomy is still controversial. Monticone et al., in a large multicenter study, reported no significant differences in terms of the biochemical and clinical response after an adrenalectomy between patients with and without contralateral aldosterone suppression and an LI ≥ 4 [72]. The clinical significance of CR < 1 in patients with a 2 < LI < 4 was investigated by Umakoshi et al. in a small cohort of 29 patients [73]. The authors reported a significant difference in terms of the overall cure rate of PA between patients with and without contralateral suppression (81% versus 31%), pointing out a possible major usefulness of CR in evaluating the “grey zone” of AVS results. On the other hand, Wolley et al. observed a greater proportion of biochemical cure and a lower number of antihypertensive drugs after an adrenalectomy in patients displaying CR < 1; however, differences between the studies in terms of the definition of lateralisation and the selection of patients could partially account for the different results [74].

In addition, some experts suggest that CR can be useful when the radiologist fails to catheterize one of the adrenal veins (more often the right adrenal vein) and the opposite site is suppressed, suggesting the possible presence of unilateral PA [75,76]. However, it must be taken into account that up to 30% of BAH patients show a suppressed non dominant A/C ratio, indicating that a CR < 1 is not specific enough to diagnose unilateral PA and refer patients for surgery [13].

More recently, El Ghorayeb et al. [56] did not observe significant differences in the blood pressure response to an adrenalectomy between patients with and without contralateral suppression; these authors suggest the use of the adrenal aldosterone/peripheral aldosterone (without cortisol correction) to predict the clinical and biochemical response to an adrenalectomy.

Finally, a plasma metanephrine measurement was recently proposed as a useful tool to evaluate the selectivity and lateralisation during AVS [71,77]. The main advantage of a metanephrine measurement is that it avoids cortisol fluctuation (metanephrine is produced continuously from epinephrine leaking from storage vesicles within adrenal medullary cells), it is not influenced by ACTH (1,24) stimulation, and the gradient between the adrenal and peripheral sample is significantly higher [77]. In fact, metanephrine is produced almost exclusively in the adrenals [78] and is rapidly cleared from the circulation, allowing a greater adrenal/peripheral ratio compared to the cortisol ratio. Furthermore, a metanephrine assay may be particularly useful in some specific situations in which a cortisol assay could give misleading information (e.g., the co-existence of subclinical hypercortisolism) [79]. In conclusion, an LI ≥ 4 should be considered diagnostic of unilateral PA in all patients, whereas an LI < 2 indicates a bilateral form. An LI comprised of between two and four can be considered as a “grey zone” and other AVS parameters (CR and IR), clinical and biochemical features, and adrenal morphology may help to reach the final decision on the preferred therapeutic approach (an example of the AVS procedure with an interpretation of hormonal data is provided as a Supplementary multimedia file).

## 10. Conclusions

The subtype diagnosis of PA is challenging and the optimal strategy is still debated. Despite being costly and invasive, AVS is still the “gold standard” test for a subtype diagnosis, as recommended by guidelines [6,80]. In the last few years, many efforts have been devoted to identifying alternative tests to substitute or to reduce the number of patients undergoing AVS. Nevertheless, none of these tests have demonstrated an adequate accuracy to substitute AVS in a significant amount of patients. Further multicentric prospective studies are warranted to identify a common and recognized AVS protocol.

## Figures and Tables

**Table 1:** Sensitivity, specificity, and accuracy of the clinical scores and alternative tests currently available to predict unilateral (or, when indicated, bilateral) PA.

Test/Clinical Score	Sensitivity	Specificity	Accuracy	Reference
Age < 40 and unilateral adrenal nodule > 1 cm with normal contralateral adrenal gland [13]	n.a.	100%	n.a.	[11]
	68%	83%	71%	[21]
	71%	n.a.	n.a.	[22]
	18%	100%	54%	[23]
Age < 35 and unilateral adrenal nodule > 1 cm with normal contralateral adrenal gland	100%	n.a.	n.a.	[22]
Typical Conn’s adenoma, serum K^+^ < 3.5 mmol/L and/or eGFR ≥ 100 mL/min/1.73 m^2^	53%	100%	74%	[23]
	46%	80%	58%	[21]
	39%	89%	56%	[24]
Typical Conn’s adenoma, serum K^+^ < 3.5 mmol/L and/or eGFR ≥ 100 mL/min/1.73 m^2^ and age < 40 years	59%	100%	68%	[21]
Serum K^+^ ≤ 3 mmol/L and PAC ≥ 25 ng/dL and/or urinary aldosterone greater ≥ 30 µg/24 h (+stage III hypertension)	32% (23%)	95% (97%)	67% (64%)	[11]
No adrenal nodule, serum K^+^ ≥ 3.5 mmol/L, ARR post-captopril < 490 ^#^	50%–67% (7 points–5 points)	100%–94% (7 points–5 points)	75%–80% (7 points–5 points)	[25]
Posture stimulation test	n.a.	n.a.	85%	[26]
	64%	70%	67%	[11]
	44%–56% (1 and 4 h respectively)	71%–75% (1 and 4 h respectively)	52%–62%	[27]
	70%	79%	75%	[28] (Torino)
	51%	78%	69%	[28] (Brisbane)
	71%	100%	41%	[29]
	35%	100%	46%	[29] *
ACTH stimulating test	91%	81%	90%	[30]
	83% (to predict BAH)	88% (to predict BAH)	84%	[31]
Steroid profiling	83%	76%	80%	[32]
Urinary 18OHF > 510 µg/24 h	35%	100%	84%	[33]
Plasma 18oxoF > 4.7 ng/dL	83%	99%	92%	[34]
Serum 18OHB > 100 ng/dL	n.a.	n.a.	82%	[26]
Serum PTH > 80 ng/L	74%	82%	76%	[35]
NP-59 scintigraphy scan	n.a.	n.a.	72%	[26]
^11^C-metomidate PET-CT	76%	87%	80%	[36]

PAC = plasma aldosterone concentration; eGFR = extimated glomerular filtration rate; ARR = aldosterone to renin ratio; PTH = parathyroid hormone; NP-59 = [6β^131^I]iodomethyl-19-norcholesterol; n.a. = not available; ^#^ Scoring system for the diagnosis of bilateral PA calculated as follows: no adrenal nodule at adrenal CT scanning, 3 points; serum K^+^ ≥ 3.5 mmol/L, 2 points; aldosterone to renin ratio (ARR) post-captopril < 490 pg/mL/ng·mL·h^−1^, 2 points; * Postural response of 18OHB was evaluated.

**Table 2:** Adrenal vein sampling indices, definition, clinical significance, and recommended cut-off in clinical practice.

Index	Measurement	Clinical Significance	Suggested Cut-Off
**Selectivity index (SI)**	Cortisol_adrenal vein_/Cortisol_peripheral vein_	Successful adrenal vein cannulation	SI > 3 for basal studies SI > 5 for ACTH (1-24) stimulated AVS
**Lateralization index (LI)**	(Aldosterone/Cortisol)_dominant adrenal vein_/(Aldosterone/Cortisol)_non dominant adrenal vein_	Lateralization of aldosterone production	LI > 4 for unilateral PA LI < 3 for bilateral PA 3 < LI < 4 grey zone
**Ipsilateral ratio (ILR)**	(Aldosterone/Cortisol)_dominant adrenal vein_/(Aldosterone/Cortisol)_peripheral vein_	Gradient of aldosterone production between the adrenal vein and a peripheral vein	ILR > 2 is required in some centers together with CLR < 1 to define unilateral PA
**Contralateral ratio (CLR)**	(Aldosterone/Cortisol)_non dominant adrenal vein_/(Aldosterone/Cortisol)_peripheral vein_	Suppression of aldosterone production in the non-dominant side	CLR < 1 can be used as additional criteria for the interpretation of suboptimal studies
**Absolute contralateral aldosterone ratio** [56]	Aldosterone_non dominant adrenal vein_/Aldosterone_peripheral vein_	Absolute suppression of aldosterone production in the non-dominant side	<1.5 predicts outcomes after adrenalectomy

## Supplementary Materials

Supplementary materials can be found at www.mdpi.com/1422-0067/18/4/848/s1.
