# Supplementary material for: Subtype Diagnosis of Primary Aldosteronism: Is Adrenal Vein Sampling Always Necessary?
Source: Int J Mol Sci. 2017 Apr 17;18(4):848. doi: 10.3390/ijms18040848 (PMC5412432; doi:10.3390/ijms18040848)
Supplement: Supplementary file 1 [file ijms-18-00848-s001.zip › Supplementary file 3.pdf]

Subtype Diagnosis of Primary Aldosteronism: is Adrenal Vein Sampling always necessary?

Fabrizio Buffolo[1], Silvia Monticone[1], Tracy A. Williams[1,2], Denis Rossato[3], Jacopo Burrello[1], Martina Tetti[1], Franco Veglio[1], Paolo Mulatero[1]

[1] Division of Internal Medicine and Hypertension Unit, Department of Medical Sciences, Via Genova 3, 10126, University of Torino, Italy.

[2] Medizinische Klinik und Poliklinik IV, Klinikum der Ludwig-Maximilians-Universität München, Munich, Germany.

[3] Service of Radiology, University of Torino, Italy

F.B. and S.M. equally contributed to this manuscript.

Corresponding author:

Paolo Mulatero, MD, Division of Internal Medicine and Hypertension, Department of Medical Sciences, University of Torino, Via Genova 3, 10126, Torino, Italy.

e-mail: paolo.mulatero@unito.it

Phone: +390116336959 Fax: +390116336931

Key words: aldosterone; primary aldosteronism; adrenal vein sampling; cosyntropin stimulation; aldosterone producing adenoma; bilateral adrenal hyperplasia

Word count: 8819 excluding references and tables.

Number of figures and tables: 2 tables, 1 supplementary multimedia file

Supplementary multimedia file

Example of sequential non stimulated AVS procedure

During AVS procedure the catheter is inserted through a femoral vein approach.

In the inferior vena cava, the first blood sample is collected for peripheral cortisol measurement.

The left adrenal vein is cannulated, and the second blood sample is collected for aldosterone and cortisol measurement.

The ratio between the left adrenal cortisol and the IVC cortisol is higher than 3, which means that the left adrenal vein has been succesfully cannulated.

The radiologist is now trying to cannulate the right adrenal vein, that drains directly into the inferior vena cava. The ratio between the right adrenal cortisol and the inferior vena cava cortisol is lower than 3, which means that the right adrenal vein was not succesfully cannulated.

In the second attempt the catheter tip is placed in the correct position and the cortisol ratio is now higher 3.

To define the side of aldosterone overproduction the cortisol corrected aldosterone ratios are calculated on each side.

In this first example we have a right aldosterone to cortisol ratio of 40 and a left aldosterone to cortisol ratio of 30.

This makes a lateralization index of 1.33 indicating bilateral aldosterone overproduction.

In this second example we have a right aldosterone to cortisol ratio of 50 and a left aldosterone to cortisol ratio of 10, resulting in a lateralization of 5.

In this case we also have a contralateral ratio < 1 which indicates a suppression of aldosterone production on the left side.

Therefore the final diagnosis is unilateral aldosterone overproduction with contralateral suppression.
